# Behavioral and neurophysiological changes associated with a single session of bimanual task practice after stroke

**DOI:** 10.1016/j.cnp.2026.05.005

**Published:** 2026-05-28

**Authors:** Joshua Jacob, Jessica Hesling-Fox, Masahiro Yamada, Shailesh Kantak

**Affiliations:** a Neuroplasticity and Motor Behavior Laboratory, Jefferson Moss Rehabilitation Research Institute, Elkins Park, PA, United States of America; b Department of Kinesiology, Whittier College, Whittier, CA, United States of America; c Pathokinesiology Laboratory, Rancho Los Amigos National Rehabilitation Center, Los Angeles, CA, United States of America

**Keywords:** Stroke, Bimanual task practice, Corticospinal excitability, Transcranial magnetic stimulation

## Abstract

**Objective:**

Stroke survivors often show slower, uncoordinated bimanual performance. This study examined the effects of a single session of bimanual task practice on performance, coordination, motor corticospinal excitability (CSE), and transcallosal inhibition in stroke survivors compared to age-matched controls.

**Methods:**

Eleven individuals with chronic stroke and ten neurotypical controls practiced a naturalistic bimanual task for 400 trials. Movement time, errors and bimanual coordination ability were assessed before and after practice. Transcranial magnetic stimulation was used to examine CSE of the ipsilesional and contralesional motor cortices (M1), and ipsilateral silent period duration and inhibition in the two M1s at pre- and post-test.

**Results:**

Both groups significantly reduced movement time without increasing errors. Controls improved bimanual coordination, but stroke survivors did not. Controls showed significant increases in bilateral CSE, while stroke survivors showed increase only in contralesional CSE. Ipsilateral silent period duration and inhibition remained unchanged in both groups.

**Conclusions:**

A single session of bimanual task practice improves speed, but not bimanual coordination in stroke survivors. Novel to this study, contralesional, but not ipsilesional CSE is modulated following a single bimanual task practice session.

**Significance:**

Findings highlight potential clinical relevance for neurorehabilitation strategies targeting bimanual coordination for improved arm function.

## Highlights

• Stroke survivors and controls improve bimanual performance speed with practice.
• Controls, but not stroke survivors improve bimanual coordination with practice.
• Bimanual practice increased contralesional, not ipsilesional, motor excitability.

## 1 Introduction

Most activities of daily living involve coordinated use of both hands to achieve functional goals (Bailey et al., 2014; Kilbreath and Heard, 2005). Following a unilateral stroke, bimanual performance is slower and clumsier, with poor spatiotemporal coordination between arms (i.e., impaired bimanual coordination). These impairments in bimanual performance and coordination arise from weakness, sensory loss and impaired control of the contralesional arm (Nguyen et al., 2023), as well as motor control deficits of the ipsilesional (less affected) arm (Johnson & Westlake, 2021a, 2021b; Schaefer et al., 2009). Stroke also disrupts interhemispheric connections and higher-level motor planning areas that influence how individuals with stroke plan and coordinate bimanual actions (Gooijers and Swinnen, 2014; Toyokura et al., 1999). As a result, bimanual performance and coordination is impaired for everyday actions and lead to greater nonuse and disability (Haaland et al., 2012; Johnson et al., 2022; Kang and Cauraugh, 2014; Kantak et al., 2017; Kantak et al., 2016; Potts and Kantak, 2023).

Although bimanual task practice is increasingly recognized as an essential component of stroke rehabilitation, relatively little is known about the behavioral and neurophysiological processes engaged in bimanual task practice in stroke survivors. Bimanual task practice is distinct from bilateral priming interventions. Bilateral priming involves rhythmic repetitive in-phase and/or anti-phase movements designed to either prepare the nervous system prior to task practice (Hsieh et al., 2017; McCombe Waller et al., 2014; Stinear et al., 2008, Stinear et al., 2014; Stoykov et al., 2022) or to improve paretic arm performance through coupling entrainment (Raghavan et al., 2017; Whitall et al., 2000). In contrast, bimanual task practice involves repetition of goal-directed functional actions that require the two hands to coordinate with each other in time and space to ensure success and efficiency (Lee et al., 2017). For example, transferring food from a bowl to a plate requires one to stabilize the plate while scooping the food from the bowl and transferring it to the plate. Such real-world task practice demands flexible interplay of different spatiotemoral coordination modes that override the natural tendency toward symmetry (Kantak et al., 2017). Despite increasing recognition of its relevance, little is known about the behavioral and neurophysiological effects of bimanual task practice in stroke survivors.

Most stroke rehabilitation research has focused on bilateral priming combined with unimanual practice. This approach has demonstrated potential benefits for the contralesional arm as well as performance of bimanual task speed (Wu et al., 2011; Wu et al., 2013). The improvements resulting from bimanual priming have been linked to changes in the excitability of the ipsilesional and contralesional motor cortices; although, findings have been inconsistent (Choo et al., 2015). For example, Lewis and Byblow (2004) reported unreliable changes in the ipsilesional motor cortex excitability (Lewis and Byblow, 2004), whereas Stinear et al. (2014) observed significant increase in ispilesional corticospinal excitability accompanied by a decrease in the contralesional corticospinal excitability (Stinear et al., 2014). Further, they also reported an increase in transcallosal inhibition (TCI) from the contralesional to ipsilesional hemisphere with bilateral priming (Stinear et al., 2014). In contrast to priming, relatively fewer studies have examined the effects of bimanual task practice on arm function and neurophysiologic outcomes after stroke. The few available studies have primarily focused on unimanual paretic arm outcomes alone (Brunner et al., 2012; Desrosiers et al., 2005; Lee et al., 2017). Only one study reported beneficial effects of symmetric bimanual force control training on improved force performance and bimanual coordination on a nonfunctional trained task in stroke survivors (Kang and Cauraugh, 2014). Thus, the effects of bimanual task-specific practice on performance and coordination of practiced bimanual task are not known in stroke survivors.

In neurotypical individuals, bimanual task practice has been shown to improve motor performance and modulate motor cortical excitability (Carson and Swinnen, 2002; Naito et al., 2021; Neva et al., 2012). Following bimanual practice, Neva et al. (2012) found faster bimanual performance with better coordination between hands. These behaviors were accompanied by increases in bilateral motor cortical excitability. Similarly, long term bimanual practice has been associated with changes in interhemispheric communication. For example, musicians trained at an early age show reduced interhemispheric inhibition (Ridding et al., 2000). While these neurophysiological adaptations to bimanual practice are evident in neurotypical individuals, the behavioral and neurophysiological effects of practice of tasks that involve interactive coordination between the two hands for real-world goals are unknown in stroke survivors.

In this study, we examined the immediate effects of a single session of naturalistic bimanual task practice in individuals with stroke compared with age-matched neurotypical adults. The task was chosen to closely mimic real-world functional actions that demand complex interactive coordination between the two arms. Our first objective was to determine whether one bout of intensive bimanual task practice would improve behavioral bimanual performance measured as movement time (MT), accuracy and ability for bimanual coordination. We hypothesized that bimanual task practice will lead to faster bimanual performance and improved ability for bimanual coordination in both stroke and control groups. Our second objective was to assess whether these behavioral changes were accompanied by neurophysiological adaptations. We hypothesized that bimanual task practice would increase ipsilesional and contralesional motor corticospinal excitability and reduce transcallosal inhibition (TCI) in stroke survivors and controls, reflecting improved interhemispheric communication in support of bimanual actions.

## 2 Methods

### 2.1 Participants

Eleven individuals with chronic stroke (STR) with mild-to-moderate motor impairments and 10 age-matched neurotypical controls (CON) were recruited from Moss Rehabilitation Research Institute's patient research registry. Based on preliminary data from four participants in stroke and control groups, showing mean changes of 20.3 + 8.1 s and 30.9 + 7 s respectively, a sample size of seven participants per group estimated a power of 80% at alpha = 0.05. All participants provided informed consent approved by Thomas Jefferson University. Inclusion criteria were (a) ability to transfer at least 10 blocks with the paretic arm in the Box and block test in one minute, (b) ability to comprehend and follow instructions (c) Mini-mental scale score > 24, (d) no evidence of hemispatial neglect tested with a line bisection test, (e) no contraindications to transcranial magnetic stimulation (TMS). Exclusion criteria included individuals with bilateral stroke, complete paralyses, infratentorial lesions, pain or stiffness in the upper extremities that may interfere with task performance.

### 2.2 Clinical measures

Participants completed standardized clinical assessments to characterize their sensorimotor and cognitive impairments. Motor testing included Upper Extremity Fugl Meyer (UEFM) scores, maximum grip strength via hand dynamometer (Jamar, Sammons-Preston-Rolyan, Bolingbrook, IL). Tactile sensation was measured with Semmes Weinstein monofilaments (Touch-Test, North Coast medical, Inc., Gilroy, CA) at the distal end of the index and thumb of bilateral hands. Executive function was assessed using the trail making tests A and B.

### 2.3 Neuroimaging and lesion data

Nine of 11 participants with stroke who participated in the Brain Behavior Relationship Research Group at MRRI had research quality MRI scans acquired at the University of Pennsylvania. Research MRI scans included whole-brain T1-weighted MR images collected on a 3 T((Seimens Trio, Erlangen, Germany: repetition time = 1620 msec, echo time = 3.87 msec, field of view = 192 × 256 mm, 1 × 1 × 1 mm voxels) scanner and were manually segmented to produce a 3-D lesion mask of 0 s and 1 s, with 1 indicating a lesioned voxel. Segmentation included both gray and white matter voxels. Thresholded, binarized lesion drawings were then warped to a 1 mm × 1 mm × 1 mm common template brain (Montreal Neurological Institute “Colin27”) using a symmetric diffeomorphic registration algorithm (Avants et al., 2008, www.picsl.upenn.edu/ANTS) to translate manual lesion segmentations to standardized space. After being transformed, lesion maps were inspected by an experienced neurologist naïve to the behavioral data, and lesion volumes (in cu. mm) were recorded. For two participants, MRI scans were not available, so their lesion location was acquired from medical records; for these two participants lesion volumes were not available.

### 2.4 Experimental design

**Fig. 1:**
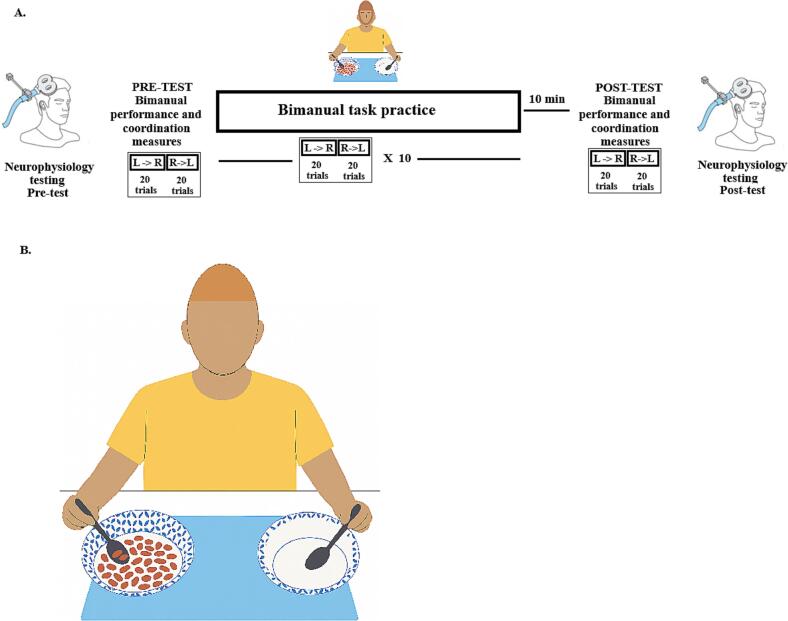
A. Experimental design: (A) Participants completed all testing in one session. Neurophysiological measures, bimanual performance and coordination were assessed before (Pre-test) and 10 min after practice (post-test). Note. L = Left, R = Right, L - > R or R - > L: The arrow indicates the direction of bean transfer. During practice, 20 trials in both directions (40 trials) were repeated 10 times, resulting in 400 acquisition trials. 1B: Task consisted of picking up two beans at a time using a spoon from one bowl, transferring them to the other spoon in midline and then transferring them to the other bowl.

Fig. 1A illustrates the timeline of the experiment. All experimental procedures were completed in a single session. After informed consent, participants completed baseline neurophysiological testing that included ipsilesional and contralesional motor corticospinal excitability (CSE) and transcallosal inhibition (TCI) between the ipsilesional and contralesional M1s. Participants also completed pre-test measures of bimanual performance and coordination of the bimanual beans transfer task. Then, participants completed bimanual task practice consisting of a total of 400 trials of beans transfer task. Ten minutes after practice ended, post-test measures of bimanual performance and coordination were obtained. The session ended with neurophysiological testing consisting of CSE and TCI between both M1s.

### 2.5 Bimanual task

The bimanual upper extremity task used in this study was a modified version of the unimanual simulated feeding task (Schaefer et al., 2015; Schaefer and Duff, 2017). Two cups (15 cm in diameter) were secured to a board 45 cm apart, placed 20 cm from the participant body (Fig. 1B). Participants were instructed to spoon up two kidney beans at a time from the ipsilateral cup with one hand, transfer the beans to a spoon held by the contralateral hand in midline and then place the beans in the contralateral cup with the other hand. The instructions prioritized speed (as fast as possible) and accuracy (without dropping the beans or carrying more than 2 beans at a time). Participants did not receive any instructions about upper extremity movement pattern or moving the two hands simultaneously at any point. One trial consisted of transferring 2 beans at a time: with one block equaling 20 repetitions transferring 40 beans in one direction. Each block began when the participants picked up the first pair of beans and ended when they finished placing the last pair of beans over to the other box. Participants completed the beans-transfer task in two consecutive blocks- one 20-trial block moving the beans from left to right bowl and another 20-trial block moving the beans from right to left.

#### 2.5.1 Pretest and post-test test

To quantify the effect of practice on motor performance, pretest and post-test test, each consisting of a 20-repetition block from right to left and another 20-repetition block from left to right were administered.

#### 2.5.2 Bimanual task practice

Participants practiced the bimanual task with a goal to improve speed and accuracy of their performance. They completed 10 repetitions of two 20-trial blocks of the bean-transfer task—first transferring beans from the left to the right bowl, followed by the reverse direction. This amounted to a total of 400 trials of beans transfer task. After each 20-trial block, participants received feedback about the MT and were told to move faster than the previous block to keep them engaged and motivated. If they accomplished the task faster than the previous block, research assistants provided reinforcing feedback with statements like “*Great job!! You beat your previous record!*” If the MT goal was not met, then the research assistant provided encouraging feedback (e.g., “*Try harder! You can move faster!*”). Participants were provided with a 2–3-min break if they reported fatigue.

### 2.6 Electromyography and brain stimulation

#### 2.6.1 Surface electromyography

Surface electrodes with preamplifiers (Motion Lab Systems- MA 411) recorded electromyographic activity (EMG) from the first dorsal interosseous (FDI) of both hands. EMG was amplified, band pass filtered (2–500 Hz), sampled at 2KHz, and recorded for off-line analyses (CED 1401 with Signal software, Cambridge Electronic Design). Maximum voluntary contraction (MVC) was recorded for each muscle prior to beginning the experiment.

#### 2.6.2 Corticospinal excitability (CSE)

Single pulse transcranial magnetic stimulation (TMS) (Magstim 200, Magstim Company Ltd., Whitland, UK) was used to obtain measures of corticospinal excitability prior to pretest and right after the post-test. CSE was assessed in the dominant/contralesional and nondominant /ipsilesional motor cortex (M1) in controls and individuals with stroke. A figure-of-eight coil attached to Magstim 200 stimulator was used to deliver TMS to the scalp overlying M1 that purportedly projected to FDI muscle. A posterior–anterior intracranial current was induced when stimulating primary motor cortex (L-M1 and R-M1, respectively) by holding the coil on the participant's scalp at 45° from the midline with the handle pointing posteriorly. Using a stereotactic neuronavigation system Brainsight (Rogue Research) with a template MRI to guide precise TMS coil position, we identified the optimal position on the scalp (hotspot) where consistent motor evoked potentials (MEPs) were evoked in the contralateral FDI muscle at the lowest stimulation intensity. The hotspot location over each M1 was marked on the neuronavigation grid to ensure consistent coil placement. Resting motor threshold was determined for each M1 as the minimum stimulation intensity that elicited a motor evoked potential (MEP) amplitude of 50 μV in at least five out of 10 consecutive trials in the relaxed contralateral FDI (Rossini et al., 2015). To quantify CSE, 20 MEPS were obtained from resting FDI of each hand by stimulating the FDI hotspot in the contralateral hemisphere with a stimulation intensity of 120% of the RMT obtained for that hemisphere.

#### 2.6.3 Transcallosal inhibition

Transcallosal inhibition from the dominant/contralesional M1 to nondominant/ipsilesional M1; and from the nondominant/ipsilesional M1 to the dominant/ contralesional M1 was measured by eliciting ipsilateral silent period (ISP). ISP is defined as a transient period of suppression of ongoing EMG activity in an isometrically contracting target muscle following suprathreshold stimulation of the ipsilateral M1(Wassermann et al., 1991). This inhibition has been shown to be transcallosally mediated via inhibitiory control (Meyer et al., 1995). To obtain ISP, twenty suprathreshold TMS pulses at 150% of RMT were delivered over M1 while the ipsilateral FDI maintained an isometric contraction at 80% of maximum EMG (Kuo et al., 2017). ISP was obtained for right and left FDI in every participant.

### 2.7 Dependent measures

#### 2.7.1 Bimanual performance and coordination

All trials during testing and practice were recorded and analyzed offline using Vimeo. Movement time for each trial was measured in seconds from the time the participant first picked up the first two beans from one bowl to them dropping the last two beans in the other bowl. Average MT of two blocks (L- > R and R- > L) was calculated for the pretest, practice and post-test test.

The number of errors were summed over two blocks (L- > R and R- > L) of pretest and post-test and included instances where beans were dropped outside the box, carrying more or less than two beans at a time, using the other hand to help.

The ability for bimanual coordination was indexed at pre-test and post-test by the percentage of trials when the two hands moved simultaneously. We rationalized that, in absence of kinematic data, the simultaneity of movements of the two hands was an estimate of bimanual coordination between hands. A grid mapping the phases of the two hands was used to systematically determine movement overlap between hands (supplementary materials)

#### 2.7.2 Neurophysiology measures

Amplified EMG signals were filtered (band-pass, 25 Hz-1 kHz), sampled at 2 kHz, and stored for offline analyses. EMG data for 3-s window prior to TMS stimulus artefact was used to measure and compare the background activity between baseline and post-test to ensure that changes in neurophysiologic measures were not due to different background activity.

*Motor corticospinal excitability (CSE):* Peak-to-peak amplitudes were measured and averaged for MEPs obtained from contralesional and ipsilesional FDI at each time point (baseline and post-test).

**Fig. 2:**
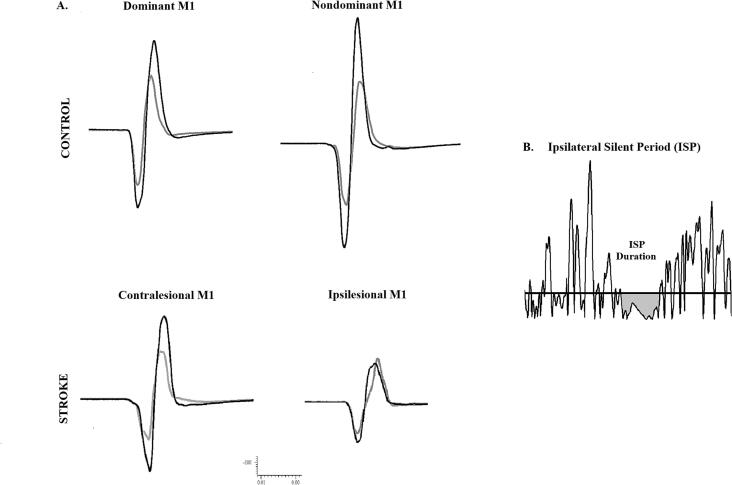
(A) Mean pre-test (gray trace) and post-test (black trace) MEP amplitudes elicited by TMS of the two M1s in representative participants from the control and stroke group. (B) Quantification of ipsilateral silent period duration and inhibition in a representative stroke participant.

*Transcallosal inhibition (TCI):* Data from twenty trials were rectified and averaged for each participant at each time. Mean baseline and standard deviation of EMG from the 100 ms window prior to the TMS pulse was calculated using custom scripts (Signal, CED, UK). TCI was indexed using two parameters: ISP duration and ISP inhibition. The onset of ISP was identified as the point after TMS pulse at which the EMG activity was suppressed at least 1 SD below the pre-TMS EMG for at least 5 ms. ISP offset was identified as the point at which the EMG activity returned to above 1SD of pre-TMS EMG. ISP duration was measured from ISP onset to ISP offset (Fig. 2B; Cunningham et al., 2015; Hayes et al., 2023; Lin et al., 2020). ISP inhibition expressed as the percentage ratio of mean EMG over the ISP duration to the mean EMG area of an identical time window just prior to the TMS pulse and calculated as [(ISP Area/ Mean preTMS EMG area)] X 100. Smaller values indicate greater inhibition (Hayes et al., 2023).

### 2.8 Statistical analysis

The Shapiro-Wilk test was used to assess the normality of the data. Non-normal data (MT, ipsilesional and contralesional MEPs and silent period duration) were log-transformed before statistical analysis. Movement time (seconds) and percentage of coordinated trials were compared using 2 group (CON, STR) X 2 time (pretest and post-test) repeated measures ANOVA with repeated measures on time. Practice related improvements in MT were analyzed using 2 group (CON, STR) X 10 blocks (practice blocks) repeated measures ANOVA with repeated measures on blocks. Average MEP amplitude of the ipsilesional and contralesional M1, TCI measures (ISP duration and inhibition) were analyzed using 2 group × 2-time repeated measures ANOVA to compare the effects of practice on the two groups. Significant interactions were followed up with post-hoc tests using Bonferroni correction. All tests were completed with SPSS-1.

## 3 Results

Eleven participants with stroke and 10 age-matched neurotypical controls completed the study. (Table 1). Table 2 describes the lesion location, lesion volume, time since stroke and Fugl-Meyer impairment score for each participant in the stroke group.

**Table 1:** Demographic and clinical data for control and stroke groups.

	Neurotypical controls	Stroke	Significance *p* values
Age	65.1 (8.65)	60.72 (11.35)	0.16
Sex (F, M)	5, 5	4, 7	
UEFM	56.63 (7.4)	–	
MMSE	28.8 (1.13)	27.81 (1.7)	0.071
Pinch strength (dominant/nonparetic)	12.09 (5.01)	9.49 (5.35)	0.133
Pinch strength (nondominant/paretic)	10.45 (6.36)	7.75 (4.79)	0.145
TMT (B-A)	29.16 (21.01)	62.69 (42.66)	0.018*

**Table 2:** Individual stroke participant descriptors of impairment, lesion characteristics and chronicity.

Stroke participant#	UEFM	Lesion location	Lesion volume (cu mm)	Time since stroke (months)
ST_1	58	Right Posterior Limb Internal Capsule	660	53
ST_2	59	Right Posterior Limb Internal Capsule	954	71
ST_3	61	Right Anterior pons	256	10
ST_4	48	Right Corona radiata	2383	121
ST_5	42	Right Subcortical white matter in MCA and ACA distribution	–	102
ST_6	50	Left frontoparietal lesion involving sensorimotor cortex and underlying white matter	124,034	89
ST_7	43	left frontal involving motor, premotor, prefrontal cortex and underlying white matter	94,677	37
ST_8	61	left parietal cortex and underlying white matter	17,371	14
ST_9	63	Left frontoparietal lesion involving sensorimotor cortex and underlying white matter	60,476	83
ST_10	65	Left parietal cortex and underlying white matter	75,835	139
ST_11	63	Left frontal cortex and underlying white matter	–	186

Individual participant data for the stroke group including Upper Extremity Fugl Meyer (UEFM) scores, lesion location, lesion volume (cu. mm) and time since stroke (in months). 3 T MRI lesions were available for all participants except ST_5 and ST_11. Lesion volume was quantified. Lesion location for these participants was extracted from medical records; thus the lesion volume data was not available for these participants.

### 3.1 Bimanual performance and coordination

**Fig. 3:**
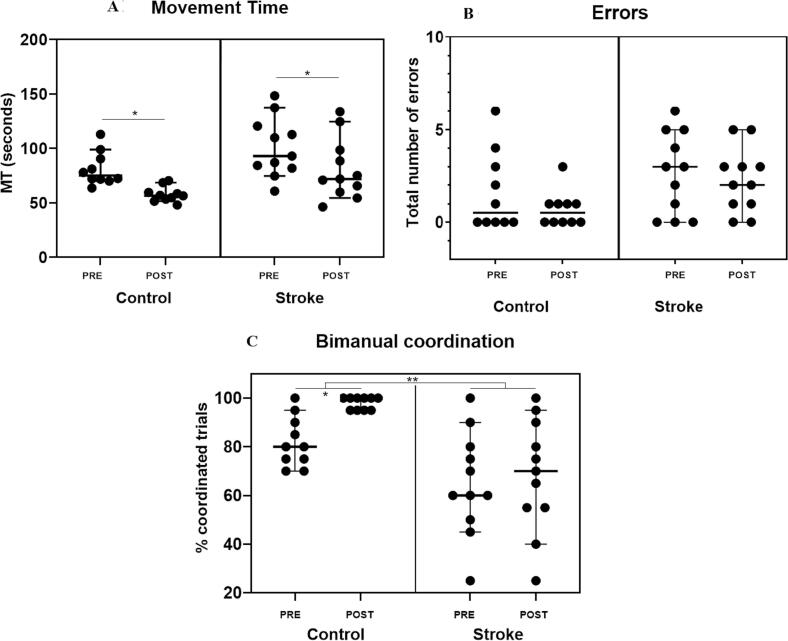
Change in (A) Mean Movement time (SD) (seconds) CON: pre-test (PRE): 81.18 + 15.29 s; post-test (POST): 57.86+ 7.01 s; STR: PRE: 101.92 + 26.81 s, POST: 80.9 + 28.02 s, (B) median number of errors (median (interquartile range): CON: 0.5 (0–3.25); POST: 0.5 (0–1); STR: PRE: 5 (3–6), POST: 3(2–5), (C) percentage of coordinated trials (interquartile range): CON: 80 (73.75–91.25); POST: 100 (95–100); STR: PRE: 60 (50–80) POST: 70 (55–90). B and C: Graphs show median with 95%confidence intervals; each dot represents individual participant). *: significant main effect of time within each group; **significant time X group interaction.

*Movement time (MT):* At baseline, the control group completed the bimanual task faster compared to the stroke group (mean pretest MT (s): CON: 81.18 + 15.29; STR: 101.92 + 26.81; t (1,19) = 4.604, *p* = 0.045). Over the course of practice, both groups improved their movement speed (main effect of practice blocks, F (1.44,27.36) = 3.859, *p* = 0.046). There was a significant main effect of group indicating that the control group was faster compared to the stroke group (F (1,19)-7.95; *p* = 0.011); however, there was no significant group X practice block interaction (*p* = 0.302). Compared to pretest, both groups moved faster at the post-test (mean post-test MT (s): CON: 57.86+ 7.01; STR: 80.9 + 28.02; main effect of time, F (1,19) = 144.05, *p* < 0.001), indicating practice benefited MT in both groups (Fig. 3A). There was no group X time interaction (*p* = 0.112).

*Error:* There was no significant change in the number of erroneous trials from pre to post (Control: pre: 1.6 + 2.1, post: 0.7 + 0.98); Stroke: (pre: 2.63 + 2.2, post: 2.27 + 1.73); *p* = 0.089, Fig. 3B). There was no significant main effect of group (*p* = 0.085) or a group X time interaction (*p* = 0.456).

*Coordination:* At baseline, the control group showed more ability for bimanual coordination with significantly more coordinated trials compared to the stroke group (t(19) = 5.026; *p* = 0.037). There was a significant time X group interaction (F(1,19) = 7.495; *p* = 0.013). Post-hoc comparisons indicated that for the control group, % coordinated trials significantly increased from 82 + 10.33% to 98 + 2.588%; while the change in the stroke group was nonsignificant from 65 + 21.21 at pretest to 68.18 + 23.27% (Fig. 3C).

### 3.2 Neurophysiological measures

**Fig. 4:**
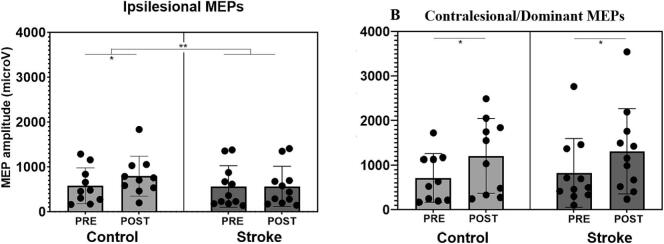
Change in mean MEP± SD amplitude evoked with stimulation of (A) Ipsilesional/nondominant M1: CON: nondominant: at pre-test (PRE): 577.19 + 400.54 μV; post-test (POST): 793.47 + 446.08 μV (*p* = 0.021); STR: PRE: 563.50 + 463.26 μV, POST: 564.96 + 446.59 μV; *p* = 0.967) and (B) Contralesional/Dominant M1: CON: PRE: 712.96+ 543.2 μV, POST: 1205 + 838.57 (*p* = 0.007) μV; Stroke: PRE: 827.61 + 772.32 μV, POST: 1308.27 + 958.43 μV; =0.001). *: significant main effect of time within each group; **significant time X group interaction.

*Ipsilesional MEP:* Fig. 4A shows average pre-test and post-test MEP amplitudes from representative participants from the control and stroke group. There was a significant group X time interaction (F(1,19) = 5.329, *p* = 0.032), indicating that the change in ipsilesional MEP from baseline to post-test was different for the two groups. For control participants, MEP amplitude of the nondominant hemisphere significantly increased from 577.19 + 400.54 μV) at baseline to 793.47 + 446.08 μV at post-test (*p* = 0.021). In contrast, there was no difference in MEP amplitude for the ipsilesional motor cortex from baseline to post-test (Baseline: 563.50 + 463.26 μV, post-test: 564.96 + 446.59 μV; *p* = 0.967; Fig. 4A).

*Contralesional MEP:* There was a significant effect of time (F (1,19) = 61.9, *p* < 0.001; Fig. 4B) indicating that both groups showed increase in MEP amplitude of the dominant/contralesional side (Controls: Pretest: 712.96+ 543.2 μV, Post- test: 1205 + 838.57 μV; Stroke: Pretest: 827.61 + 772.32 μV, Post- test: 1308.27 + 958.43 μV). However, there was no significant main effect of group (*p* = 0.742) or group X time interaction (*p* = 0.859).

**Fig. 5:**
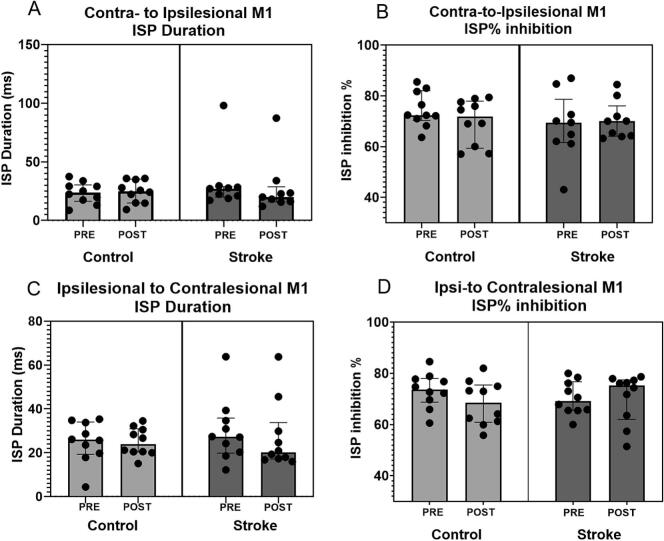
Change in ISP duration in ms for TCI from (A) contralesional to ipsilesional M1 (median (interquartile range): CON: PRE: 23.81 (16.39–30.37) ms, POST: 24.92 (14.97–35.22) ms; STR: PRE:27.10 (19.94–28.93) ms, POST (19.97 (15.87–28.7) ms; and (C) ipsilesional to contralesional M1 (median (interquartile range): CON: PRE: 25.85 (19.24–33.99) ms, POST: 23.90 (20.3–30.93) ms; STR: PRE:27.20 (19.8–35.78) ms, POST (20.05 (17.10–33.73) ms; and % ISP inhibition for TCI from (B) contralesional to ipsilesional M1 (median (interquartile range): CON: PRE: 72.39 (70.31–82.03), POST: 70.83 (59.34–77.91); STR: PRE: 69.48 (61.61–78.66), POST (69.99 (64.12–76.04); and (D) ipsilesional to contralesional M1 (median (interquartile range): CON: PRE: 73.69 (68.73–77.99), POST: 68.54 (60.9–75.44); STR: PRE: 69.18 (65.49–76.69), POST (75.21 (61.98–77.41). Data show median with interquartile range, each dot represents individual participant.

*Transcallosal inhibition from contralesional to ipsilesional M1:* The duration ISP from the contralesional to ipsilesional motor cortex reduced in the stroke group compared to controls, however, the interaction between group and time was not significant (F(1,19) = 3.18; *p* = 0.092; Fig. 5A). There was no significant effect on time (*p* = 0.263) or group (0.581). Similarly, for ISP inhibition from contralesional to ipsilesional motor cortex, there was no significant group by time interaction (1,17) = 2.51; *p* = 0.131), or main effect of time (0.54) or group (*p* = −0.44; Fig. 5C).

*Transcallosal inhibition from ipsilesional to contralesional M1:* For ISP duration and inhibition from ipsilesional to contralesional M1, there was no significant group X time point interaction (ISP duration: F(1,19) = 0.686, *p* = 0.418; ISP inhibition: F(1, 19) = 1.545, *p* = 0.23) or significant effect of group (ISP duration: *p* = 0.552; ISP inhibition: *p* = 0.865) or time point (ISP duration: *p* = 0.916; ISP inhibition: *p* = 0.265; Fig. 5B and D).

## 4 Discussion

We examined the effects of a single session of bimanual task practice on performance, bimanual coordination, bilateral motor CSE and TCI in chronic stroke survivors and age-matched neurotypical controls. With practice, both groups improved the speed of bimanual performance without increasing errors. Contrary to our hypothesis, task practice did not significantly improve the ability for bimanual coordination in stroke survivors. Ipsilesional corticospinal excitability did not increase with bimanual practice in the stroke group. In contrast, contralesional corticospinal excitability increased following practice. In contrast to our hypothesis, a single bimanual practice session had no effect on ipsilateral silent period duration and inhibition.

### 4.1 Performance and coordination changes with bimanual task practice

Our results corroborate prior studies demonstrating improvements in speed and accuracy associated with bimanual task practice in chronic stroke survivors (Doost et al., 2019; Gerardin et al., 2022). In the present study, performance speed significantly increased accompanied by a nonsignificant reduction in errors in both groups. While we did not systematically analyze speed-accuracy tradeoff, these findings indicate an improvement in skill performance with a single bout of intense practice. Despite the baseline differences between the two groups, both groups improved their performance speed to a similar extent. Prior work has demonstrated that stroke survivors learn unimanual skills to a similar extent to age-matched controls (Hardwick et al., 2017; Mooney et al., 2020). Here, our work extends those findings to bimanual skills, supporting the benefits of task practice. The patients in the present study had mild motor and cognitive impairments; thus, it remains to be examined if these findings can be generalized to those with moderate to severe motor and cognitive impairments. Prior work has shown that those with more severe deficits engage their paretic arm only during bimanual actions (Chen et al., 2023), making it more relevant to test the effects of bimanual practice across a larger sample with different levels of motor and cognitive deficits in the future.

Contrary to our hypothesis, the stroke group did not significantly change the ability for bimanual coordination with practice. This finding contrasts with those of Doost and Gerardin who showed significant practice-driven improvements in bimanual coordination in stroke survivors (Doost et al., 2019; Gerardin et al., 2022). Both the Doost and Gerardin studies involved multiple days of practice, in contrast to a single practice session in the present study. Further, and more importantly, practice in Doost and Gerardin study directly trained coordination between arms. In contrast, the practice in our study focused predominantly on speed and accuracy without direct feedback or instructions on simultaneity or coordination of the two arms during practice. Thus, our findings in the context of previous work highlight the specificity of information/instruction provided during practice. Providing specific feedback or reinforcing simultaneity or interlimb coordination during practice may provide a better learning signal to improve bimanual coordination (Kantak et al., 2017).

### 4.2 Motor corticospinal excitability and transcallosal inhibition changes with bimanual task practice

Similar to previous studies (Neva et al., 2012), we found that a single bout of bimanual task practice increased motor CSE in neurotypical controls. However, in the presence of unilateral stroke, the increase in ipsilesional motor corticospinal excitability was blunted. Prior work investigating the motor cortical excitability effects of rhythmic, and mostly symmetric bilateral movements have yielded mixed results for ipsilesional motor cortex. While rhythmic active-passive bilateral therapy for 4 weeks was accompanied by increase in ipsilesional M1 excitability (Stinear et al., 2014), similar rhythmic intervention (Stinear and Byblow, 2004) or bimanual task practice (Lewis and Byblow, 2004; Summers et al., 2007) did not have consistent effects on ipsilesional excitability or map characteristics. It is likely that a single session of bimanual practice may be insufficient in driving enough plasticity in the lesioned cortex; and more intense or higher dose of practice may be needed to increase ipsilesional excitability. Previous studies reporting changes in ipsilesional motor CSE investigating task practice employed multiple days of practice (Kantak et al., 2018). Alternately, our assessment of motor CSE using one suprathreshold intensity of 120% RMT may not have been as sensitive as using a stimulus-response curve measuring MEP amplitudes at a range of TMS intensities (Kantak et al., 2018).

Bimanual task practice increased contralesional motor CSE. This finding corroborates with previous work indicating the role of contralesional motor cortex in bimanual learning. For example, suppressing the contralesional motor cortex excitability resulted in detriments of bimanual performance in stroke survivors (Takeuchi et al., 2012). Finally, in contrast to our hypothesis, we did not find any consistent effects of bimanual task practice on the duration or the amount of inhibition of the ipsilateral silent period. Transcallosal communication is crucial for bimanual motor performance (Morishita et al., 2022; Naito et al., 2021). In chronic stroke survivors with mild-to-moderate motor impairments, transcallosal inhibition from the contralesional M1 to ipsilesional M1 has been implicated in unilateral motor impairment (Harris-Love et al., 2011, Harris-Love et al., 2016; Murase et al., 2004); however, the evidence is mostly contrary or inconclusive at best (Cunningham et al., 2015; Fokas et al., 2025; Gerges et al., 2022; Lin et al., 2020). Thus, the relationship of TCI to motor recovery in general, and bimanual control and coordination, in particular, is more complex. In neurotypical individuals, transcallosal inhibition was related to the synchrony of in-phase symmetrical bimanual performance (Bortoletto et al., 2021). However, its role in complex bimanual actions that involve different types of coordination is not clear. Our findings suggest that a single bout of bimanual task practice may be insufficient in driving consistent changes in TCI in both neurotypical controls and stroke. Future research needs to investigate how TCI relates to distinct bimanual actions and their long-term practice.

### 4.3 Limitations

The present preliminary study has some limitations. Our sample was individuals with mild motor impairments with relatively high UEFM scores. However, learning bimanual actions is crucial, since bimanual control and coordination may be impaired even in those with mild motor impairments. Our measure of bimanual coordination (number of trials with simultaneous movements of the two arms) is a less sensitive measure of bimanual coordination than kinematic/kinetic measures. Further, we did not test long-term retention of benefits acquired through bimanual task practice. Finally, we limited the neurophysiological assessments to motor cortical excitability and TCI due to time constraints; thus, limiting our ability to infer mechanisms (e.g., short-interval cortical inhibition or intracortical facilitation) underlying the changes in motor corticospinal excitability.

## 5 Conclusions

Post-stroke individuals at the chronic stage with mild-to-moderate deficits exhibited similar bimanual movement time improvements as age-matched controls. While controls demonstrated improvement for the ability for bimanual coordination, more practice or directed feedback on moving the arms simultaneously may be needed in stroke survivors to improve bimanual coordination. Ipsilesional motor corticospinal excitability and transcallosal inhibition between the two motor cortices was not modulated after bimanual task practice in stroke survivors. However, the contralesional motor corticospinal excitability increased following task practice, similar to controls. Future investigations investigating the role of a broader neural network including premotor cortex, supplementary motor cortex and cerebellum in bimanual skill learning are needed to inform neuromodulatory interventions to improve bimanual function after stroke.

## CRediT authorship contribution statement

**Joshua Jacob:** Methodology, Writing – original draft. **Jessica Hesling-Fox:** Methodology. **Masahiro Yamada:** Methodology, Writing – review & editing. **Shailesh Kantak:** Conceptualization, Methodology, Writing – review & editing, Supervision, Funding acquisition.

## Funding source

This work was supported by Eunice Kennedy Shriver National Institute of Child Health and Human Development (NICHD), National Institutes of Health, Grant #R01HD092481 to Kantak SS.

## Declaration of competing interest

The authors declare that they have no competing financial interests or personal relationships that could have appeared to influence the work reported in this paper.
