# Supplementary material for: Behavioral and neurophysiological changes associated with a single session of bimanual task practice after stroke
Source: Clin Neurophysiol Pract. 2026 May 28;11:393–402. doi: 10.1016/j.cnp.2026.05.005 (PMC13264191; doi:10.1016/j.cnp.2026.05.005)
Supplement: Supplementary file 1 — Supplementary material [file mmc1.docx]

**Supplemental material 1 A:**

Rubric for capacity for bimanual coordination: The dark cells indicate the areas that typically won’t be occurring simultaneously. For example, while transferring the beans from one spoon to the other, the two hands will be in the transfer phase. So, the overlap will be occurring during the empty cells. For each trial, mark the overlap if occurring. If not, keep the cell blank.

|  |  | **PICKUP HAND** |  |  |  |
| --- | --- | --- | --- | --- | --- |
| Transport from midline to bowl | Pick up beans | Transport to the midline for transfer | Transfer |  |  |
|  |  |  |  | Transfer |  |
|  |  |  |  | Transport from midline to drop bowl | **DROP HAND** |
|  |  |  |  | Drop beans |  |
|  |  |  |  | Transport to midline to get beans |  |

**Supplemental material 1B:**

**MEP Latency:**

*Dependent measure:*

For each of recorded EMG traces, MEP latency was measured as the time elapsed between the TMS and beginning of the MEP trace. This time reflects the time for excitation of the cortical neurons, conduction of the pyramidal tract and summation of the descending volleys at the spinal level and the conduction time of peripheral axons. Briefly, MEP latency was scored manually by a trained, but blinded research assistant using a custom-made script that displayed raw and rectified EMG traces. One cursor was automatically placed at TMS onset, and the second cursor was manually positioned at the MEP onset, defined as the point where the trace for MEP inflected from the baseline. For 37 EMG traces out of 840 traces (4%), the MEP onset could not be reliably determined; hence those trials were excluded from the analysis.

*Analysis:*

Average latency was calculated for each participant for the MEPs yielded by ipsilesional/nondominant stimulation and contralesional/dominant M1 stimulation. The latency data was non-normal; hence, it was log-transformed prior to analyses and then plotted with raw data. For the MEP latency of contralesional/dominant arm and ipsilesional/nondominant stimulation, separate 2 group (controls, stroke) X 2 time (pretest and post-test) repeated measures ANOVA with repeated measures on time was used.

*Results:*

Please see figure below. For the ipsilesional/nondominant M1 stimulation, we observed a significant main effect of group (F (1,19) = 10.41; p=0.004), indicating that for the stroke group, ipsilesional stimulation yielded MEPs with significantly longer latencies than the control group. There was no effect of time (p=0.119) or group X time interaction (p=0.154). For contralesional/dominant M1 stimulation, there was no significant main effect of group (p=0.71) or time (p=0.774) or group X time interaction (p=0.714).


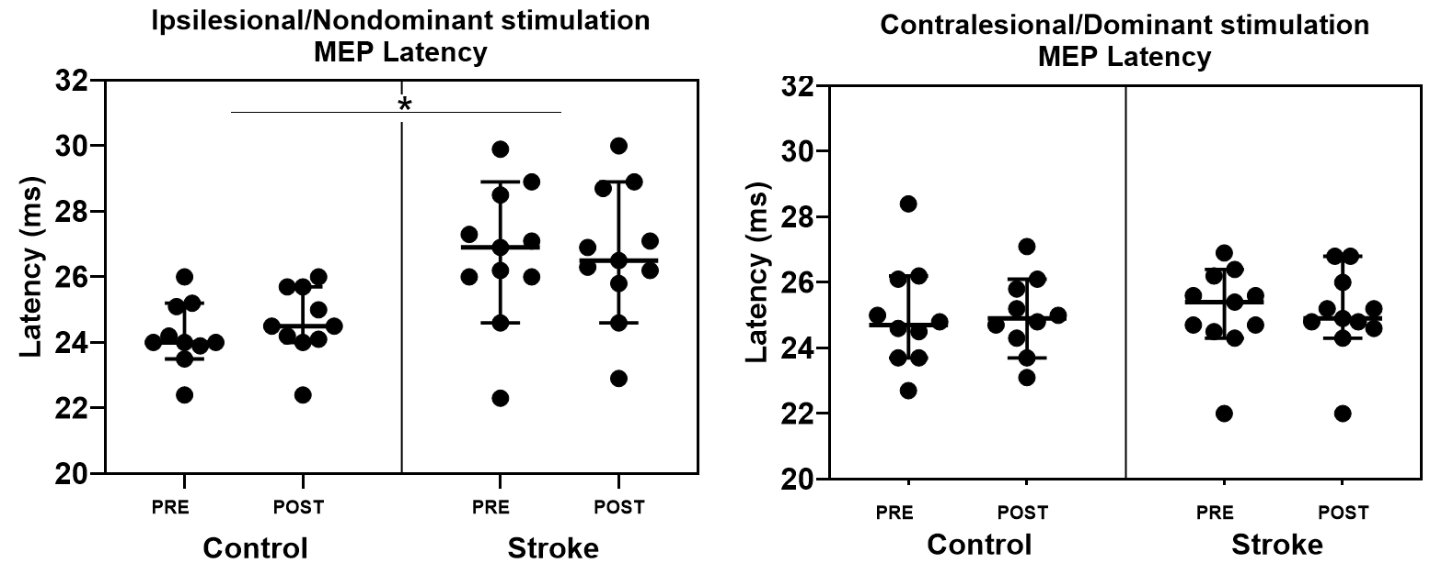


The above figure shows the change in median MEP latency with 95%ile from PRE to POST practice. For the MEPs evoked by stimulation of the ipsilesional/nondominant M1, latency in ms (median (IQR): CON: PRE: 24 (23.8-25.13), POST: 24.5 (24.08-25.7); STR: PRE: 26.9 (26-28.5), POST: 26.5 (25.8-28.7). For MEPs evoked by stimulation of the contralesional/dominant M1, latency in ms (median (IQR): CON: PRE: 24.7 (23.7-28.4), POST: 24.9 (24.15-25.88); STR: PRE: 25.4 (24.5-26.2), POST: 24.9 (24.6-26).
